# A multicenter, open-label, phase III study of Abcertin in Gaucher disease: Erratum

**DOI:** 10.1097/MD.0000000000012066

**Published:** 2018-08-17

In the article, “A multicenter, open-label, phase III study of Abcertin in Gaucher disease”,^[1]^ which appears in Volume 96, Issue 45 of *Medicine*, Table 1 contained incorrect information and the correct table appears below.

**Table 1:**
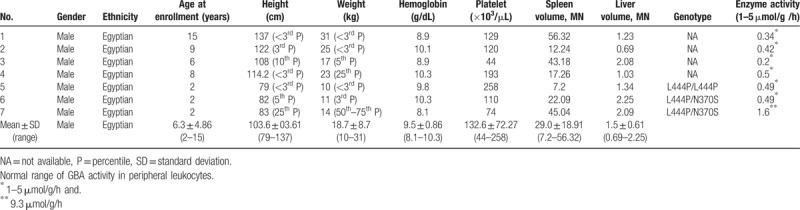
Demographics and Baseline Clinical Characteristics of the Per Protocol Population.
